# Epigenetic modifiers either individually or in specific combinations impair viability of patient-derived glioblastoma cell line while exhibiting moderate effect on normal stem cells growth

**DOI:** 10.21203/rs.3.rs-2698139/v1

**Published:** 2023-03-20

**Authors:** Arshak Alexanian, Heidi Stoellinger, Virginea De Araujo Farias, Alfredo Quinones-Hinojosa

**Affiliations:** Cell Reprogramming & Therapeutics LLC; Cell Reprogramming & Therapeutics LLC; Mayo Clinic; Mayo Clinic

**Keywords:** glioblastoma, epigenetics, stem cells, cell viability

## Abstract

Glioblastomas (GBM), also known as glioblastoma multiforme, are the most aggressive type of brain cancers. Currently, there is no real treatment for GBM and thus there is a compelling need for new therapeutic strategies for such type of cancers. Recently, we demonstrated that specific combinations of epigenetic modifiers significantly affect the metabolism and proliferation rate of two most aggressive GBM cell lines D54 and U-87. Importantly, these combinations exhibited minimal effect on normal stem cells growth. In this study we demonstrated that the combinations of modulators of histone and DNA covalent modifying enzymes that synergistically suppress D54 and U87 cell lines growth, also impair the viability of a patient freshly-derived GBM stem cell line. These data suggest that epigenetic modifiers alone or in specific combinations exhibit cytotoxic effect on established and low passage patient derived GB cell lines and thus could be a promising therapeutic approach for such type of brain cancers.

## Introduction

Glioblastomas (GBM) are an aggressive type of cancers that can occur in the brain and/or spinal cord, with a median survival of 12.6 months GBM [1]. GBM originates from glial cells, are highly heterogeneous and create complex interactions with cells both within and surrounding the tumor [2]. This make it very difficult to treat and a cure is challenging. Current treatment options include chemotherapy, radiation, and surgical removal [3], however, these treatments may only slow progression of the cancer and/or reduce some signs and symptoms. Thus, there is an urgent need for new therapeutic options for this deadly disease.

Over decades, basic and preclinical cancer research has been based on the use of established, commercially available cell lines, originally derived from patients’ samples but adapted to grow indefinitely in specific culture conditions [4]. The most common established cell lines being used in GBM experimental models are U251, U87, U373MG, D54 and T98G cell lines [5–7]. However, none of these cell lines were able to faithfully recapitulate GBM, since long-term culturing of these and other GBM cell lines lead to a large number of accumulated genomic alterations. These mutations distance the cell lines from their original GBM tissue characteristics and in number of cases makes them unsuitable for biomarker discovery, drug screening, and therapeutic preclinical testing [8]. Despite this, different types of cancer lines, including some of the GBM cell lines have been extremely useful for shedding light on cancer cell biology [7, 9].

The effort to find preclinical models able to better predict the clinical outcome led to the generation of patient-derived cancer models [10]. Currently, the gold-standard for preclinical models are serum-free, low-passage cell lines derived from primary tumors [11]. Although extremely useful, the patient-derived models of GBM should be used in parallel with established models to create a more complete image of the true complexity of GBM [4].

In our previous study we showed that specific combinations of histone methyltransferases inhibitors (BIX01294 and DZNep), histone deacetylases inhibitor (Trichostatin A) and DNA methyltransferase inhibitor in relatively small concentrations suppress the growth of D54 cells, but exhibited minimal effect on bone marrow derived mesenchymal stem cells (BM-MSCs) proliferation [6]. Based on these discoveries we decided to test whether the same combination of epigenetic modifiers can exhibit similar effect on another well-known glioma cell line such as U87. Results showed that while individually the DZNep, TSA and BIX01294 at their low concentrations showed a moderate effect on the viability of U87 cells, in combinations they exhibited a synergistic effect [12], as demonstrated for D54 cells. Importantly, these combinations exhibited minimal or moderate effect on adipose-derived mesenchymal stem cells (AD-MSCs) growth, as demonstrated previously for BM-MSCs [6]. These results also showed that the most effective combination was the medium concentrations of TSA + BIX01295 that almost completely killed U87 cells demonstrated with the MTT test and cell count by trypan blue [12].

Thus, all our recent studies showed that revealed combinations of epigenetic modifiers exhibited similar cytotoxic effect on two different GBM cell lines. These data led us to test whether these unique combinations will affect also a patient derived glioblastoma stem cell line. To this end, we tested the most effective combinations of chromatin modifying agents on patient derived cell line such as GBM965 developed by Dr.Quinones-Hinojosa group [13–19].

## Materials And Methods

Expansion of Patient-derived Glioma. GBM965 glioma cell line was derived, characterized, and cultured as previously described [13–19]. For these studies cryopreserved GBM965 cells were thawed and grown in DMEM/F-12 media (Gibco, Grand Island, NY, USA) supplemented with, 10% Gem21 NeuroPlex^™^ Supplement without Vitamin A (Gemini, West Sacramento, CA, USA), 20ng/ml bFGF (R&D Systems, Minneapolis, MN, USA) and 1% Pen/Strep (Gibco, Grand Island, NY, USA).

Expansion of MSCs. Fat tissue was obtained from the Medical College of Wisconsin’s Tissue Bank (patient remaining anonymous). For purification of MSCs from pre-adipocytes and other cell types, fat tissue was digested in a 1% Collagenase (Gibco, Grand Island, NY, USA) solution for 1 h then centrifuged at 50 g for 1 min. The pellet containing AD-MSCs was washed three times in 1x PBS. These cells were then plated in 25-cm^2^ culture flasks in α-MEM supplemented with 10% FBS, 1% Pen/Strep, and 1% GlutaMAX and incubated at 37°C in 5% CO_2_. The medium was changed every 4 days until the cells reached confluence.

Treatments with chromatin modifying agents.

Cells were seeded in 96 and 24-well plastic plates at a density of 15000/cm^2^. After 4 h of attachment, cells were exposed to combinations of different concentrations of three epigenetic modifiers. The following concentrations were used for each modulator in low and medium doses: BIX01294–100nM and 1uM (Cayman Chemicals, Ann Arbor, MI, USA); Trichostatin A (TSA) – 10nM, 50nM (Cayman Chemicals, Ann Arbor, MI, USA). The following concentrations were used for RG108 (Sigma-Aldrich, St. Louis, MO, USA) in low, medium, and high doses: 50uM, 100uM, and 500uM.

Cell viability assay with MTT. Cells grown in 96-well-plates for 120 h were tested for viability with the MTT assay as described previously (35). Briefly, MTT tetrazolium salt (5 mg/mL) (Invitrogen, Eugene, OR, USA) was added to each well, and incubated for 4 h at 37°C. The formazan crystals resulting from mitochondrial enzymatic activity on the MTT substrate were solubilized with 12mM SDS (Liberty Scientific, Lisle, IL, USA). Absorbance was measured at 570 nm using a microplate reader (Accuris Instruments, Edison, NJ, USA). Cell survival was expressed as absorbance relative to that of untreated controls.

Cell Count. For cell count, cells grown in 24 well plates for 120 h were collected and counted using trypan blue stain. Control and each treatment was carried out in triplicate.

### Statistical Analysis.

All data was expressed as mean ± SEM for the number (n-3) of independent experiments performed. Differences among the means for all experiments described were analyzed using one-way analysis of variance. Newman-Keul’s post hoc analysis was employed when differences were observed by analysis of variance testing (p < 0.05).

## Results

In this study we aimed to investigate, whether the specific combinations of epigenetic modulators, that had demonstrated synergistic activity on U87 and D54 cells would exhibit a similar effect on patient-derived GB cell line (GBM965). To this end, different combinations of BIX01294, TSA and RG108 at (conditionally called) small (S), medium (M) and high (H) concentrations that exhibited the highest cytotoxic effect on U87 and D54 cell lines but showed minimal effect on normal stem cells were tested on GBM965 cells growth. We also tested small, medium and high concentrations of RG-108 individually, on GBM965 cells growth. For cell viability assays (metabolism and proliferation) the MTT test and trypan blue cell count approaches were used.

**Figure 1.**
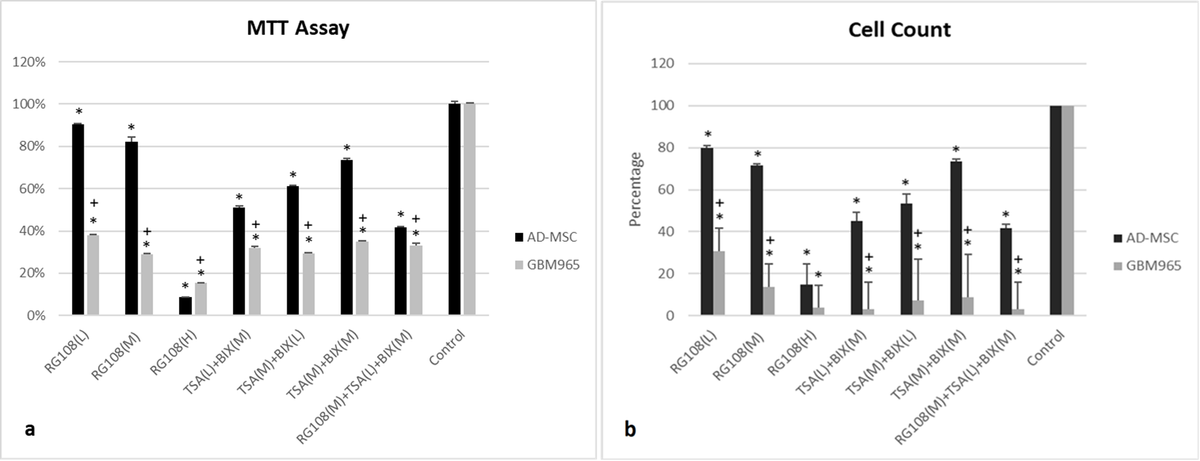
MTT cell metabolism assay (a) and cell count analysis (b) were used to study the effects of different epigenetic modifiers (BIX01294, TSA and RG108) and their combinations on AD-MSCs and GBM965 cells viability. Asterisk are comparison of treated samples with control, plus sign are comparison of AD-MSCs with GBS960. P ≤ 0.05 was considered significant

Results showed that while individually the RG108 at the high concentration (500 uM) had significant effect on GBM965 cell viability it also displayed a significant effect on normal stem cells (such as AD-MSCs) proliferation and metabolic activity (Fig. 1.a,b). Medium and low concentrations of Rg108 significantly suppressed the metabolism and proliferation of GBM965 but showed minimal suppressive effect on AD-MSCs growth. (Fig. 1.a,b). Our previous studies demonstrated that RG108, at all three concentrations demonstrated only minor effect on the growth of established cell lines such as D54 and U87 [6, 12].

Similar to the previous studies on U87 and D54 cells [6, 12], the TSA and BIX01294 in combinations, at different concentration such as TSA(L) + BIX(M), TSA(M) + BIX(L) and TSA(M) + BIX(M) significantly suppress GBM965 cell metabolism and proliferation while showed little or moderate effect on AD-MSC viability (Fig. 1.a,b). Combination of RG108 + TSA + BIX affect AD-MSCs growth without significantly augmenting the cytotoxic effect of TSA + BIX on GBM965 cells.

## Discussion

One of the strategies to develop new therapeutics for GBs is the use of established GB cell lines such as U251, U87, U373MG, D54,T98G and others. However, recent several studies showed that the cell lines only partially reflect the gene expression profiles of tumors found in GB patients. In addition, with long term passaging these cell lines undergo spontaneous mutation which further change their gene expression and properties. Thus, freshly developed, or low passaged glioblastoma cell lines could be more clinically relevant cellular models for basic research and drug screening.

Recently, we demonstrated that specific combinations of modulators of chromatin covalent modifying enzymes significantly affect the viability of two most aggressive glioblastoma cell lines such as D54 and U-87.

With this study we aimed to test the effect of these specific combinations of epigenetic modifiers in patient derived glioblastoma cell line. Results demonstrated that combination of two histone covalent modifiers that significantly suppress D54 and U-87 cell lines viability exhibit also cytotoxic effect on patient derived glioblastoma cell line GBM965. These studies revealed that the combination of TSA(M) + BIX(M) displayed the highest cytotoxic effect on GBM965 while exhibiting the lowest effect on AD-MSCs metabolism and proliferation. These studies also showed that DNA methyl transferase inhibitor which individually at high, medium and low concentrations exhibit only minor effect on D54 and U87 cells viability, significantly suppress GBM965 cells growth. Importantly, low and medium concentrations of RG108 only slightly affect AD-MSCs growth. Adding RG108 to TSA + BIX affect AD-MSCs growth without significantly augmenting the cytotoxic effect of TSA + BIX on GBM965 cells.

These data suggest that epigenetic modifiers alone or in specific combinations can exhibit high cytotoxicity against established and low passage patient derived glioblastomas while displaying minor effect on normal-stem cells growth and thus could be a promising therapeutic approach for such type of brain cancers.

## Data Availability

The datasets generated during and/or analysed during the current study are available from the corresponding author on reasonable request.
